# Relationship Between Pre-operative Blood Glucose Level and Length of Hospital Stay in Patients With Renal Cell Carcinoma Undergoing Laparoscopic Nephrectomy

**DOI:** 10.3389/fsurg.2021.659365

**Published:** 2021-05-24

**Authors:** Ting He, Weidong Zhu, Chunying Wang, Haowen Lu, Tiange Wu, Kehao Pan, Shuqiu Chen, Bin Xu, Weipu Mao, Wei Li, Ming Chen

**Affiliations:** ^1^Department of Urology, Zhongda Hospital, Southeast University, Nanjing, China; ^2^Surgical Research Center, Institute of Urology, Southeast University Medical School, Nanjing, China; ^3^Department of Urology, Nanjing Lishui District People's Hospital, Zhongda Hospital Lishui Branch, Southeast University, Nanjing, China; ^4^Department of Nursing, Zhongda Hospital, Southeast University, Nanjing, China; ^5^Department of Urology, Shanghai Tenth People's Hospital, School of Medicine, Tongji University, Shanghai, China

**Keywords:** renal cell carcinoma, pre-operative blood glucose, length of stay, laparoscopic nephrectomy, dose-response analysis

## Abstract

**Purpose:** The aim of this study was to assess the effect of pre-operative blood glucose (POBG) levels on the length of stay (LOS) in patients with renal cell carcinoma (RCC) undergoing laparoscopic nephrectomy.

**Methods:** We collected clinical data on 338 patients with RCC who underwent laparoscopic nephrectomy between 2014 and 2019. Univariate and multivariate logistic regression and dose-response analysis curves of restricted cubic spline function were used to investigate the relationship between POBG and LOS.

**Results:** According to the level of POBG, we divided the patients into three groups: <4.94 mmol/L group, 4.94 to <7.11 mmol/L group, and ≥7.11 mmol/L group. According to the dose-response analysis curves, we found that the adjusted risk of LOS > 2 weeks and LOS > 3 weeks gradually increased with increasing POBG. In addition, we found that among all patients, patients with POBG levels ≥ 7.11 mmol/L had a 115% higher risk of LOS > 2 weeks than patients with POBG levels <4.94 mmol/L [adjusted odds risk (aOR) 2.15; 95% CI 1.11-4.20; *p* = 0.024] and patients with POBG levels ≥ 7.11 mmol/L had a 129% higher risk of LOS > 3 weeks than patients with POBG levels <4.94 mmol/L (aOR 2.29; 95% CI 1.16-4.52; *p* = 0.017). Moreover, similar results were observed in the most subgroups analysis.

**Conclusion:** We found that in patients with RCC undergoing laparoscopic nephrectomy, higher POBG levels were significantly associated with prolonged LOS.

## Introduction

Renal cell carcinoma (RCC) is a common malignancy of the urinary system originating from the kidney, accounting for 80-85% of renal malignancies, and its incidence has been increasing in recent years (1). According to statistics, more than 400,000 new cases and more than 170,000 deaths were diagnosed worldwide in 2018 (2). Approximately 25-30% of patients initially diagnosed with RCC are reported to have developed distant metastasis (3). Partial or radical nephrectomy is the main treatment for localized RCC, while the common treatments for advanced RCC are combination therapies such as immunotherapy and molecular therapy (4).

The reasons for the increase in the incidence of RCC on the one hand is the progress of imaging examination, on the other hand, it the increased exposure to risk factors in the population. Both epidemiological and basic studies suggest that metabolic factors such as obesity, hypertension, diabetes, and dyslipidemia may affect the occurrence and development of RCC (5–7). Studies have found that elevated fasting blood glucose and diabetes are risk factors for cancer development and death in multiple organs, including the kidney (8, 9).

A growing number of studies have found that pre-operative blood glucose (POBG) has adverse effects on patients undergoing surgery. A retrospective study of 4,025 patients who underwent appendectomy and 4,266 patients who underwent laparoscopic cholecystectomy found that higher POBG levels were significantly correlated with longer length of stay (LOS) (10). In addition, one study found that non-diabetic patients with higher POBG had a higher 1-year mortality rate than diabetic patients with similar blood glucose levels when underwent elective non-cardiac surgery (11). We retrospectively investigated the clinical data of RCC patients in our hospital to determine the relationship between POBG levels and LOS in RCC patients undergoing laparoscopic nephrectomy.

## Patients and Methods

### Patients Selection

The Department of Urology of Zhongda Hospital has established a clinical database of urinary malignancies. In this study, we retrospectively collected the clinicopathological data of 354 patients with RCC who underwent radical or partial laparoscopic nephrectomy in our hospital from January 2014 to December 2019. The methodology of this study followed the criteria outlined in the Helsinki Declaration (revised in 2013) and has been ethically approved by the Ethics Committee and Institutional Review Board of Zhongda Hospital (ZDKYSB077). All the patients or their relatives who participated in this study understood and signed the informed consent form.

The inclusion criteria were as follows: (a) Radical or partial laparoscopic nephrectomy; (b) Age over 18 years. The exclusion criteria were as follows: (a) Patients with other malignant tumors (*n* = 6); (b) Patients with incomplete or missing follow-up data (*n* = 7); (c) Patients with other disease-related conditions that significantly affect survival time (*n* = 3). The final study cohort included 338 patients.

### Blood Glucose Measurement

The main point of interest in this study was the fasting glucose level 2 days before surgery or closest to the time of surgery. Plasma was collected in the morning from all patients and the same testing equipment was used. According to the manufacturer's instructions, a fully automated particle-enhanced immunoturbidimetric assay was used on the Architect ci 16200 system (Abbott Lab, Illinois, USA) and plasma glucose levels were measured using the hexokinase enzymatic method. Information on standard biosecurity and institutional safety procedures were followed throughout the blood collection process.

### Data Collection and Follow-Up

Information on patients' clinical and pathological data was obtained from hospital electronic medical records, including gender (male and female), age (≤65 years and >65 years), body mass index (BMI) (<25 and ≥25 kg/m^2^), hypertension (no and yes), diabetes (no and yes), cardiovascular disease (no and yes), smoking (no and yes), ASA score (1-2 and 3-4), Clavien-Dindo complications (I, II, and III), surgery type (partial nephrectomy and radical nephrectomy) laterality (left and right), AJCC stage (I, II, III, and IV), T stage (T1, T2, T3, and T4), N stage (N0 and N1), M stage (M0 and M1), Fuhrman grade (I, II, III, and IV), estimated blood loss (EBL) and operation duration. Two experienced surgeons performed the surgery mainly. All post-operative patients were followed up regularly by professional staff on an outpatient or telephone basis, and the survival status or cause of death was collected at each follow-up. The primary end-point of the study was the follow-up cut-off time (December 2020) or time to death. Survival time was defined as the time from the end of surgery to the end of follow-up or death. The predominant outcome was hospital LOS. LOS was defined as the interval between the date of discharge and the date of hospitalization.

### Statistical Analysis

Continuous variables were expressed as median and interquartile range (IQR), and categorical variables were expressed as *n* (%). The categorical variables were analyzed by χ^2^ test. For continuous variables, the *t*-test for slope was used in the generalized linear model. Hospital LOS was treated as a dichotomous variable (≤2 weeks or >2 weeks and ≤ 3 weeks or >3 weeks). Univariate and multivariate logistic regression were used to evaluate the relationship between POBG and hospital LOS > 2 weeks or >3 weeks. In multivariate logistic regression, we constructed three models to assess the association between POBG and hospital LOS and calculated adjusted odds ratios (aOR) and 95% confidence intervals (CI). In the basic model, we adjusted age, sex, and BMI. Subsequently, we further adjusted hypertension, diabetes, cardiovascular disease, smoking, ASA score, and Clavien-Dindo complications in the core model. Finally, in the extended model, we adjusted the tumor-related variables, such as surgery type, laterality, AJCC stage, T stage, N stage, M stage, and Fuhrman grade.

The restricted cubic spline function is a powerful tool for describing dose-response relationships between continuous variables and outcomes (12). In this study, we used restricted cubic spline function to characterize the dose-response relationships between POBG and hospital LOS >2 weeks or >3 weeks, and adjusted for the variables in the extended model. We also estimated the aOR and 95% CI for hospital LOS >2 weeks or >3 weeks corresponding to a specific POBG (13). In addition, we performed the same analysis for subgroups with proportions > 50% to determine the relationship between POBG and hospital LOS in the subgroups. SPSS software (version 24.0) and RStudio software (version 1.2.5033) were used for the analysis of this study and *P* < 0.05 was considered statistically significant.

## Results

The demographic and clinicopathological characteristics of patients in this study were shown in Table 1. By trisecting POBG levels, we divided all patients into three groups: <4.94 mmol/L group, 4.94 to <7.11 mmol/L group, and ≥4.94 mmol/L group. We found that there were statistical differences in BMI, hypertension, diabetes, cardiovascular disease, and LOS among the three groups of patients. We found higher proportions of female (28.3%), hypertension (52.2%), diabetes (32.7%), cardiovascular disease (20.4%), smoking (22.1%), ASA score 3-4 (8.0%), complications (5.3%), right side (53.1%), AJCC III/IV stage (21.2.7%), T3-4 stage (18.5%), N1 stage (5.3%), M1 stage (6.2%), LOS > 2 weeks (79.6%), and LOS > 3 weeks (38.9%) at higher POBG level (≥4.94 mmol/L) group than in the other two groups. In addition, we also observed the post-operative blood glucose levels and found a positive correlation between post-operative blood glucose levels and POBG (*r* = 0.3577, *P* < 0.001) (Supplementary Figure 1).

**Table 1:** Baseline characteristics by pre-operative glucose level in patients undergoing nephrectomy.

**Characteristic**	**All patients**	**Nephrectomy (*****n*** **= 338)**			***P*-value**
		**Glucose <4.94**	**Glucose 4.94 to <7.11**	**Glucose ≥ 7.11**	
	**No. (%)**	**No. (%)**	**No. (%)**	**No. (%)**	
Total patients	338	112	113	113	
Age, years	57.0 (50.0, 66.0)	55.0 (46.0, 65.8)	58.0 (51.5, 67.0)	58.0 (51.0, 65.0)	0.076
Age categorized, years					0.731
≤ 65	251 (74.3)	84 (75.0)	81 (71.7)	86 (76.1)	
>65	87 (25.7)	28 (25.0)	32 (28.3)	27 (23.9)	
Gender					0.257
Male	222 (65.7)	70 (62.5)	71 (62.8)	81 (71.7)	
Female	116 (34.3)	42 (37.5)	42 (37.2)	32 (28.3)	
BMI, kg/m^2^	24.6 (22.3, 27.0)	23.6 (20.9, 25.8)	25.3 (23.6, 27.4)	25.1 (23.0, 27.7)	<0.001
BMI categorized, kg/m^2^					0.005
<25	184 (54.4)	75 (67.0)	53 (46.9)	56 (49.6)	
≥25	154 (45.6)	37 (33.0)	60 (53.1)	57 (50.4)	
Hypertension					0.045
No	189 (55.9)	72 (64.3)	63 (55.8)	54 (47.8)	
Yes	149 (44.1)	40 (35.7)	50 (44.2)	59 (52.2)	
Diabetes					<0.001
No	283 (83.7)	107 (95.5)	100 (88.5)	76 (67.3)	
Yes	55 (16.3)	5 (4.5)	13 (11.5)	37 (32.7)	
Cardiovascular diseases					0.012
No	295 (87.3)	102 (91.1)	103 (91.2)	90 (79.6)	
Yes	43 (12.7)	10 (8.9)	10 (8.8)	23 (20.4)	
Smoking					0.103
No	281 (83.1)	93 (83.0)	100 (88.5)	88 (77.9)	
Yes	57 (16.9)	19 (17.0)	13 (11.5)	25 (22.1)	
ASA score					0.216
1-2	321 (95.0)	108 (96.4)	109 (96.5)	104 (92.0)	
3-4	17 (5.0)	4 (3.6)	4 (3.5)	9 (8.0)	
Clavien-Dindo complications					0.671
None	327 (96.7)	110 (98.2)	110 (97.3)	107 (94.7)	
I	5 (1.5)	1 (0.9)	1 (0.9)	3 (2.7)	
II	5 (1.5)	1 (0.9)	2 (1.8)	2 (1.8)	
III	1 (0.3)	0 (0.0)	0 (0.0)	1 (0.9)	
EBL, mL	40 (30, 100)	50 (30, 100)	40 (30, 100)	50 (30, 100)	0.567
Operation duration, min	160 (125, 204)	160 (120, 210)	150 (120, 195)	180 (150, 205)	0.569
Surgery type					0.764
Partial nephrectomy	185 (54.7)	60 (53.6)	65 (57.5)	60 (53.1)	
Radical nephrectomy	153 (45.3)	52 (46.4)	48 (42.5)	53 (46.9)	
Laterality					0.792
Left	167 (49.4)	56 (50.0)	58 (51.3)	53 (46.9)	
Right	171 (50.6)	56 (50.0)	55 (48.7)	60 (53.1)	
AJCC stage					0.326
I	252 (74.6)	83 (74.1)	84 (74.3)	85 (75.2)	
II	19 (5.6)	9 (8.0)	6 (5.3)	4 (3.5)	
III	44 (13.0)	11 (9.8)	19 (16.8)	14 (12.4)	
IV	23 (6.8)	9 (8.0)	4 (3.5)	10 (8.8)	
T stage					0.918
T1	256 (75.7)	84 (75.0)	85 (75.2)	87 (77.0)	
T2	22 (6.5)	9 (8.0)	8 (7.1)	5 (4.4)	
T3	51 (15.1)	16 (14.3)	18 (15.9)	17 (15.0)	
T4	9 (2.7)	3 (2.7)	2 (1.8)	4 (3.5)	
N stage					0.464
N0	326 (96.4)	109 (97.3)	110 (97.3)	107 (94.7)	
N1	12 (3.6)	3 (2.7)	3 (2.7)	6 (5.3)	
M stage					0.424
M0	322 (95.3)	106 (94.6)	110 (97.3)	106 (93.8)	
M1	16 (4.7)	6 (5.4)	3 (2.7)	7 (6.2)	
Fuhrman grade					0.379
I	53 (15.7)	20 (17.9)	16 (14.2)	17 (15.0)	
II	213 (63.0)	62 (55.4)	78 (69.0)	73 (64.6)	
III	64 (18.9)	27 (24.1)	18 (15.9)	19 (16.8)	
IV	8 (2.4)	3 (2.7)	1 (0.9)	4 (3.5)	
Glucose, mmol/L	5.64 (4.94, 7.11)	4.75 (4.55, 4.94)	5.64 (5.37, 5.91)	8.18 (7.11, 10.21)	<0.001
LOS, days	17.0 (14.0, 22.0)	16.0 (13.0, 20.0)	17.0 (14.5, 21.0)	28.0 (15.5, 41.0)	<0.001
LOS > 2 weeks	246 (72.8)	71 (63.4)	85 (75.2)	90 (79.6)	0.018
LOS > 3 weeks	87 (25.7)	21 (18.8)	22 (19.5)	44 (38.9)	<0.001
Survival time (months)	29.0 (16.8, 45.3)	30.5 (16.3, 49.5)	29.0 (17.0, 47.5)	28.0 (15.5, 41.0)	0.097
**Complications**					
Hematuria	3 (0.9)				
Infection	4 (1.2)				
Fever	3 (0.9)				
Renal failure	1 (0.3)				

*Continuous variables were presented as the median and interquartile range (IQR) and categorical variables were expressed as n (%). For categorical variables, P-values were analyzed by χ2 test. For continuous variables, the t-test for slope was used in generalized linear models*.

*BMI, Body mass index; AJCC, American Joint Committee on Cancer; LOS, Length of stay; EBL, estimated blood loss*.

**Figure 1:**
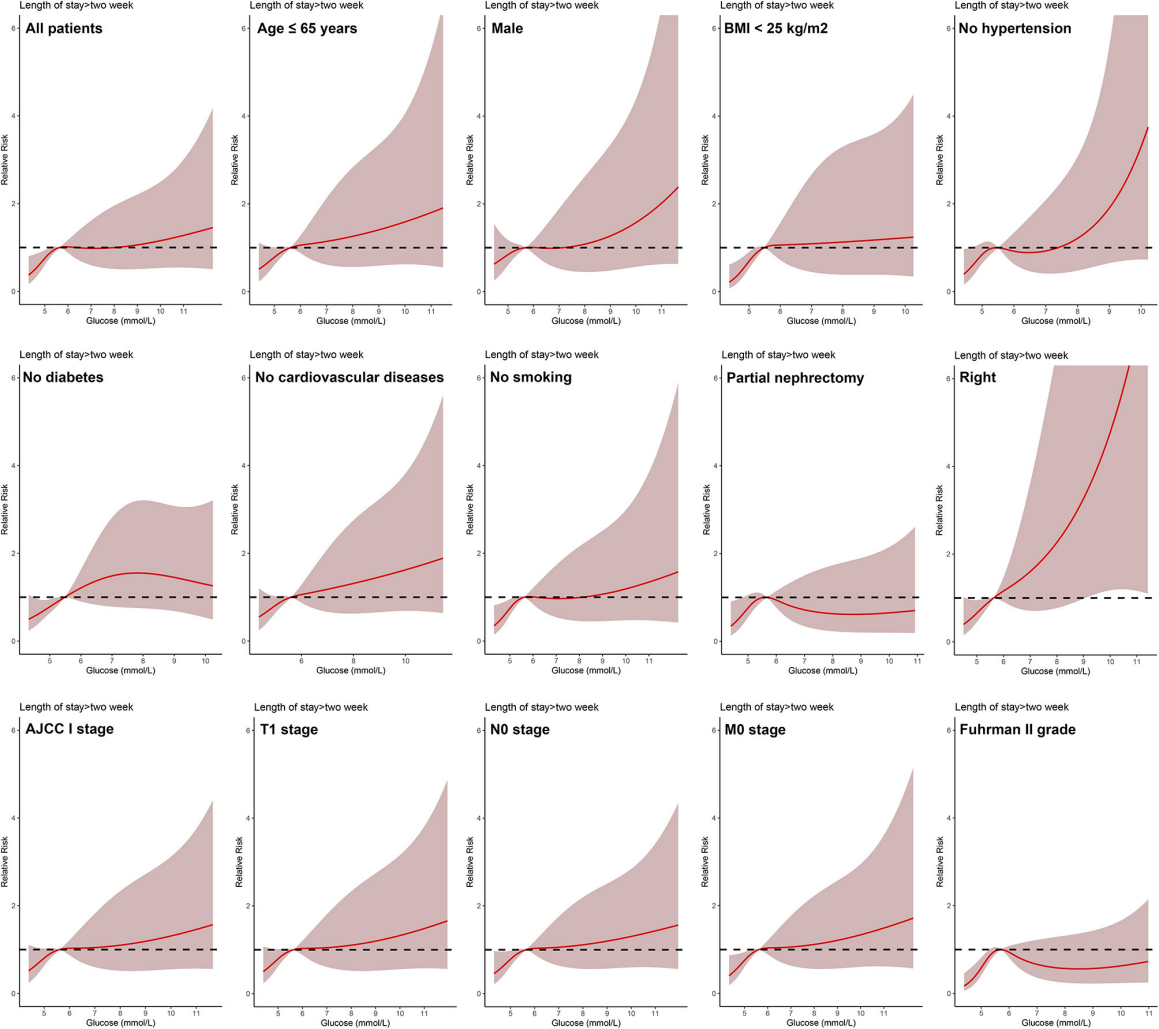
Relative risk for a hospital length of stay > 2 weeks according to POBG level. The solid black lines represent aORs based on restricted cubic splines for POBG level. The shaded areas represent upper and lower 95% CIs. Adjustment factors are the same as those in extended model of Table 4.

**Figure 2:**
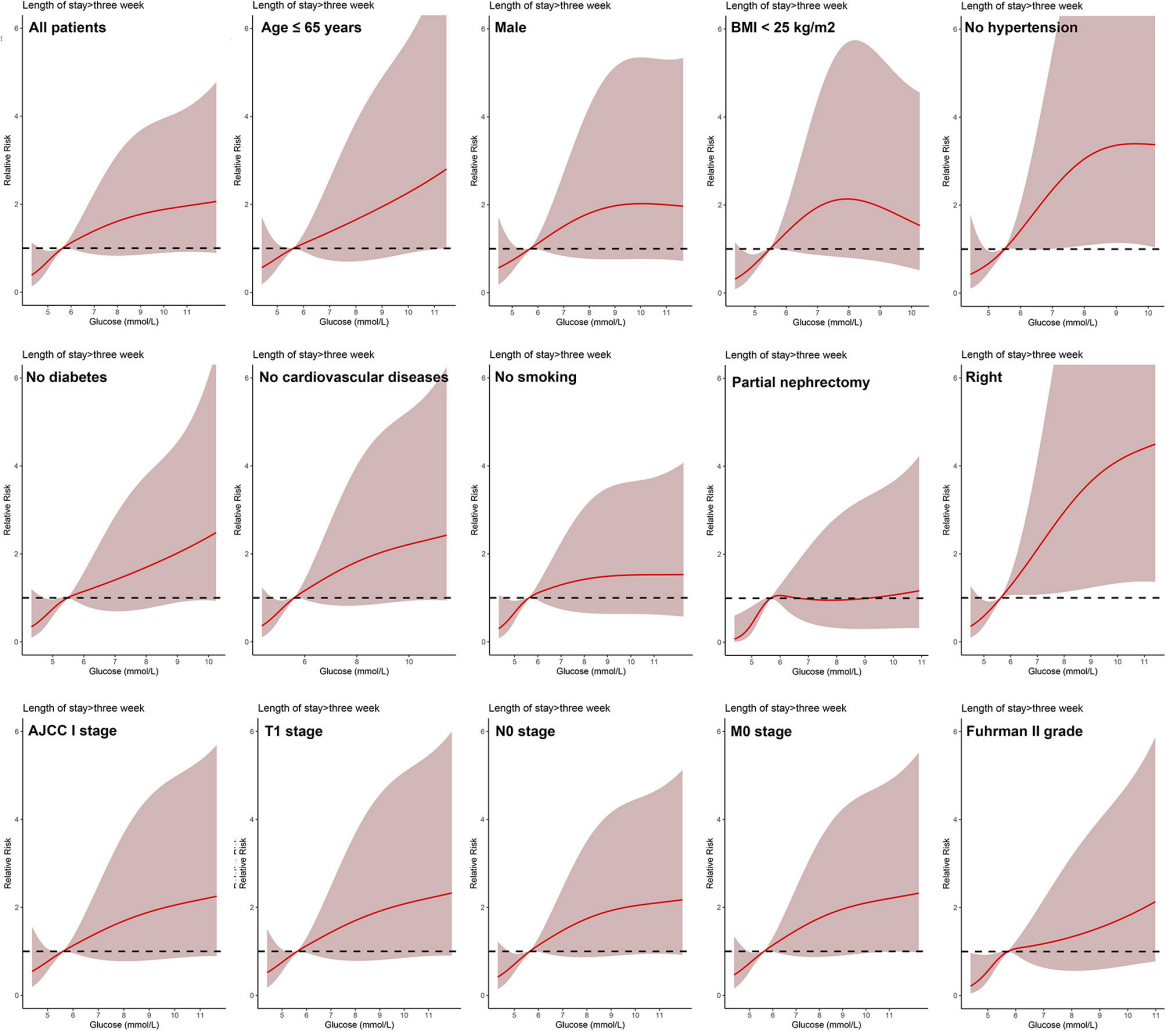
Relative risk for a hospital length of stay > 3 weeks according to POBG level. The solid black lines represent aORs based on restricted cubic splines for POBG level. The shaded areas represent upper and lower 95% CIs. Adjustment factors are the same as those in extended model of Table 4.

In all patients, after adjusting for sex, age, BMI, hypertension, diabetes, cardiovascular diseases, smoking, ASA score, Clavien-Dindo complications, surgery type, laterality, AJCC stage, T stage, N stage, M stage, and Fuhrman grade, the dose-response analysis curves of restricted cubic spline function showed that the risk of LOS > 2 weeks and LOS > 3 weeks increased with the increase of POBG (Figures 1, 2 and Tables 2, 3). In addition, the optimal POBG level was 5.66 mmol/L when the OR was 1.

**Table 2:** Weighted odds ratio and 95% confidence intervals of LOS > 2 weeks by levels of POBG levels.

**LOS > 2 weeks**	**5 mmol/L**	**6 mmol/L**	**7 mmol/L**	**8 mmol/L**	**9 mmol/L**	**10 mmol/L**	**11 mmol/L**
Overall	0.74 (0.59-0.93)	1.02 (0.87-1.18)	0.99 (0.60-1.65)	1.01 (0.52-1.96)	1.08 (0.51-2.23)	1.16 (0.54-2.51)	1.28 (0.55-3.02)
Male	0.84 (0.60-1.17)	1.00 (0.86-1.18)	0.99 (0.53-1.88)	1.08 (0.45-2.62)	1.27 (0.48-3.36)	1.58 (0.57-4.42)	2.01 (0.63-6.49)
Age ≤ 65 years	0.79 (0.61-1.01)	1.06 (0.88-1.27)	1.14 (0.62-2.12)	1.26 (0.56-2.84)	1.41 (0.59-3.36)	1.59 (0.62-4.06)	1.80 (0.59-5.51)
BMI <25 kg/m^2^	0.71 (0.55-0.91)	1.06 (0.71-1.60)	1.09 (0.45-2.67)	1.13 (0.39-3.31)	1.17 (0.38-3.59)	1.23 (0.36-4.22)	-
No hypertension	0.85 (0.65-1.11)	0.92 (0.62-1.36)	0.93 (0.41-2.10)	1.22 (0.48-3.08)	1.90 (0.65-5.58)	3.34 (0.73-15.20)	–
No diabetes	0.78 (0.64-0.95)	1.22 (0.90-1.65)	1.48 (0.79-2.81)	1.55 (0.75-3.21)	1.45 (0.68-3.08)	1.30 (0.54-3.14)	–
No cardiovascular diseases	0.81 (0.65-1.02)	1.06 (0.88-1.28)	1.18 (0.67-2.09)	1.31 (0.63-2.75)	1.46 (0.66-3.23)	1.63 (0.69-3.81)	1.81 (0.66-4.91)
No smoking	0.72 (0.55-0.94)	1.01 (0.85-1.20)	0.97 (0.54-1.73)	0.99 (0.46-2.15)	1.07 (0.45-2.51)	1.19 (0.47-2.98)	1.34 (0.47-3.83)
Partial nephrectomy	0.77 (0.56-1.06)	0.94 (0.77-1.14)	0.71 (0.34-1.50)	0.63 (0.23-1.73)	0.62 (0.20-1.90)	0.65 (0.20-2.14)	0.71 (0.20-2.63)
Right	0.65 (0.44-0.95)	1.16 (0.91-1.46)	1.60 (0.71-3.62)	2.27 (0.77-6.68)	3.28 (0.99-10.92)	4.75 (1.17-19.18)	7.01 (1.16-42.29)
AJCC I stage	0.81 (0.64-1.02)	1.03 (0.88-1.21)	1.05 (0.60-1.83)	1.10 (0.52-2.33)	1.19 (0.52-2.72)	1.31 (0.55-3.12)	1.46 (0.57-3.75)
T1 stage	0.79 (0.62-1.01)	1.03 (0.89-1.20)	1.05 (0.61-1.81)	1.10 (0.52-2.33)	1.20 (0.52-2.74)	1.33 (0.56-3.16)	1.48 (0.58-3.81)
N0 stage	0.79 (0.64-0.98)	1.03 (0.89-1.20)	1.06 (0.64-1.76)	1.11 (0.56-2.19)	1.20 (0.57-2.51)	1.30 (0.60-2.84)	1.43 (0.60-3.41)
M0 stage	0.76 (0.61-0.95)	1.04 (0.89-1.21)	1.06 (0.63-1.77)	1.12 (0.56-2.22)	1.21 (0.57-2.57)	1.34 (0.61-2.95)	1.49 (0.62-3.60)
Fuhrman II grade	0.57 (0.40-0.80)	0.93 (0.81-1.08)	0.66 (0.36-1.22)	0.57 (0.25-1.32)	0.57 (0.23-1.43)	0.63 (0.24-1.66)	0.73 (0.25-2.14)

*The following covariates were adjusted: age, gender, BMI, hypertension, diabetes, cardiovascular diseases, smoking, ASA score, Clavien-Dindo complications, surgery type, laterality, AJCC stage, T stage, N stage, M stage, and Fuhrman grade*.

*BMI, Body mass index; AJCC, American Joint Committee on Cancer; LOS, Length of stay*.

**Table 3:** Weighted odds ratio and 95% confidence intervals of LOS > 3 weeks by levels of POBG levels.

**LOS > 3 weeks**	**5 mmol/L**	**6 mmol/L**	**7 mmol/L**	**8 mmol/L**	**9 mmol/L**	**10 mmol/L**	**11 mmol/L**
Overall	0.69 (0.51-0.94)	1.12 (0.99-1.26)	1.39 (0.86-2.23)	1.61 (0.83-3.11)	1.77 (0.85-3.67)	1.88 (0.90-3.94)	1.96 (0.92-4.18)
Male	0.74 (0.49-1.11)	1.13 (0.98-1.30)	1.51 (0.84-2.71)	1.81 (0.77-4.23)	1.98 (0.76-5.14)	2.03 (0.77-5.35)	2.00 (0.75-5.30)
Age ≤ 65 years	0.79 (0.56-1.10)	1.11 (0.93-1.33)	1.37 (0.73-2.57)	1.66 (0.71-3.89)	1.95 (0.78-4.90)	2.27 (0.89-5.77)	2.62 (0.98-7.00)
BMI <25	0.67 (0.51-0.88)	1.39 (0.97-2.00)	1.95 (0.86-4.40)	2.13 (0.80-5.72)	1.96 (0.71-5.38)	1.61 (0.56-4.66)	–
No hypertension	0.68 (0.48-0.95)	1.45 (1.00-2.12)	2.34 (1.01-5.43)	3.05 (1.08-8.60)	3.36 (1.14-9.90)	3.38 (1.07-10.68)	–
No diabetes	0.75 (0.58-0.97)	1.15 (0.83-1.60)	1.41 (0.69-2.85)	1.70 (0.76-3.80)	2.01 (0.89-4.54)	2.39 (0.94-6.04)	–
No cardiovascular diseases	0.68 (0.49-0.94)	1.16 (0.96-1.41)	1.52 (0.84-2.75)	1.82 (0.82-4.00)	2.04 (0.87-4.78)	2.21 (0.93-5.25)	2.36 (0.95-5.86)
No smoking	0.66 (0.47-0.93)	1.11 (0.95-1.31)	1.29 (0.74-2.26)	1.42 (0.66-3.06)	1.49 (0.64-3.49)	1.52 (0.63-3.64)	1.53 (0.62-3.74)
Partial nephrectomy	0.43 (0.23-0.83)	1.06 (0.87-1.29)	0.98 (0.44-2.16)	0.96 (0.32-2.84)	0.99 (0.30-3.30)	1.07 (0.32-3.69)	1.18 (0.32-4.26)
Right	0.58 (0.35-0.95)	1.27 (1.06-1.52)	2.11 (1.07-4.14)	2.96 (1.13-7.72)	3.65 (1.23-10.79)	4.10 (1.33-12.66)	4.40 (1.37-14.10)
AJCC I stage	0.77 (0.57-1.05)	1.13 (0.96-1.32)	1.44 (0.82-2.53)	1.69 (0.78-3.69)	1.89 (0.80-4.50)	2.05 (0.85-4.97)	2.17 (0.89-5.34)
T1 stage	0.75 (0.54-1.05)	1.12 (0.97-1.30)	1.44 (0.83-2.50)	1.70 (0.78-3.68)	1.91 (0.80-4.56)	2.08 (0.85-5.08)	2.21 (0.89-5.46)
N0 stage	0.71 (0.53-0.95)	1.14 (0.99-1.32)	1.48 (0.89-2.45)	1.74 (0.87-3.51)	1.93 (0.89-4.15)	2.04 (0.93-4.45)	2.11 (0.95-4.70)
M0 stage	0.74 (0.55-0.98)	1.15 (0.99-1.33)	1.48 (0.89-2.45)	1.76 (0.87-3.53)	1.96 (0.91-4.24)	2.10 (0.96-4.60)	2.20 (0.99-4.88)
Fuhrman II grade	0.57 (0.35-0.93)	1.07 (0.91-1.27)	1.18 (0.63-2.19)	1.33 (0.56-3.20)	1.54 (0.59-4.00)	1.81 (0.68-4.82)	2.13 (0.78-5.87)

*The following covariates were adjusted: age, gender, BMI, hypertension, diabetes, cardiovascular diseases, smoking, ASA score, Clavien-Dindo complications, surgery type, laterality, AJCC stage, T stage, N stage, M stage, and Fuhrman grade*.

*BMI, Body mass index; AJCC, American Joint Committee on Cancer; LOS, Length of stay*.

**Figure 3:**
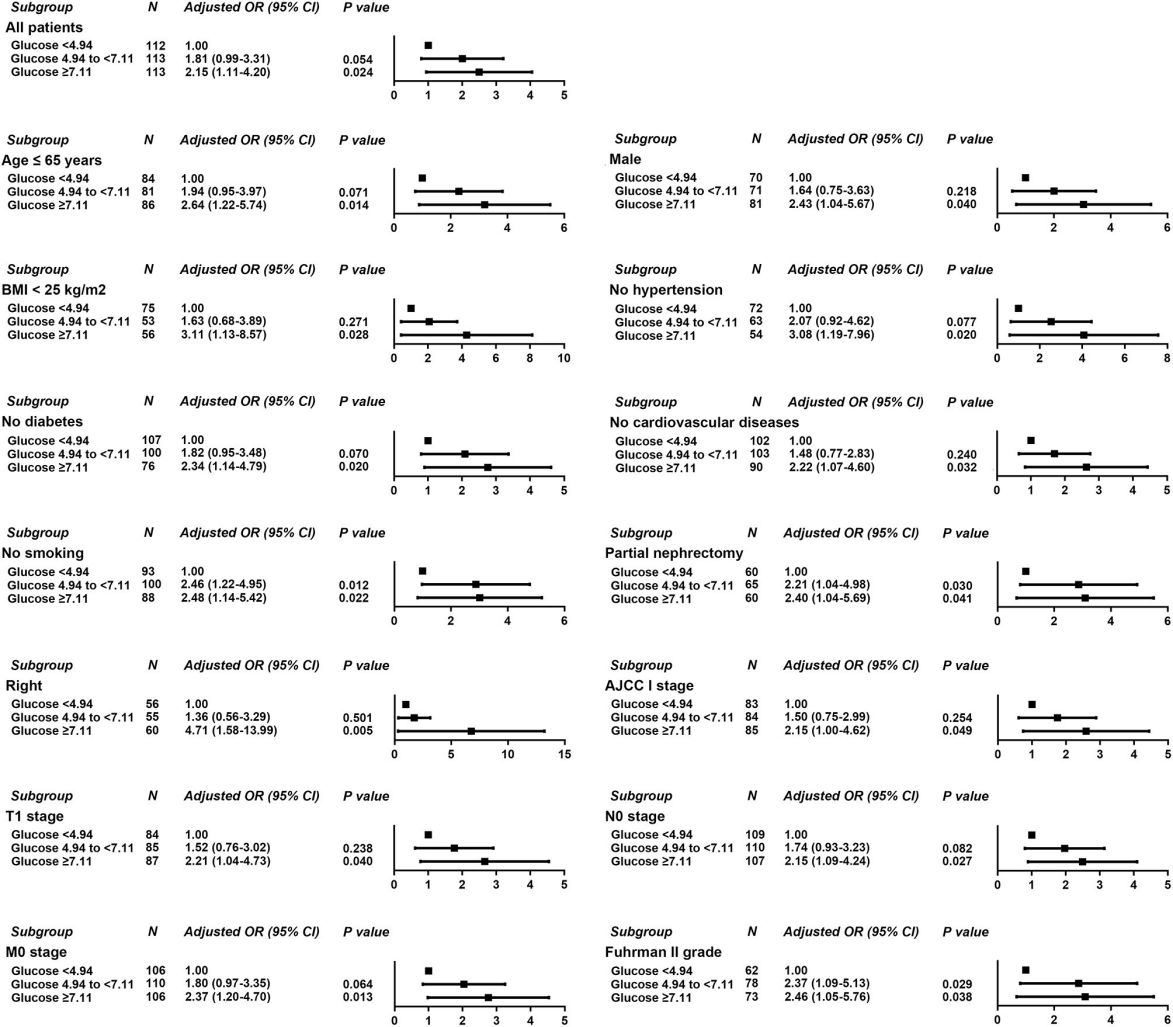
Results of subgroup analyses of POBG and a hospital length of stay > 2 weeks according to clinical characteristics.

**Figure 4:**
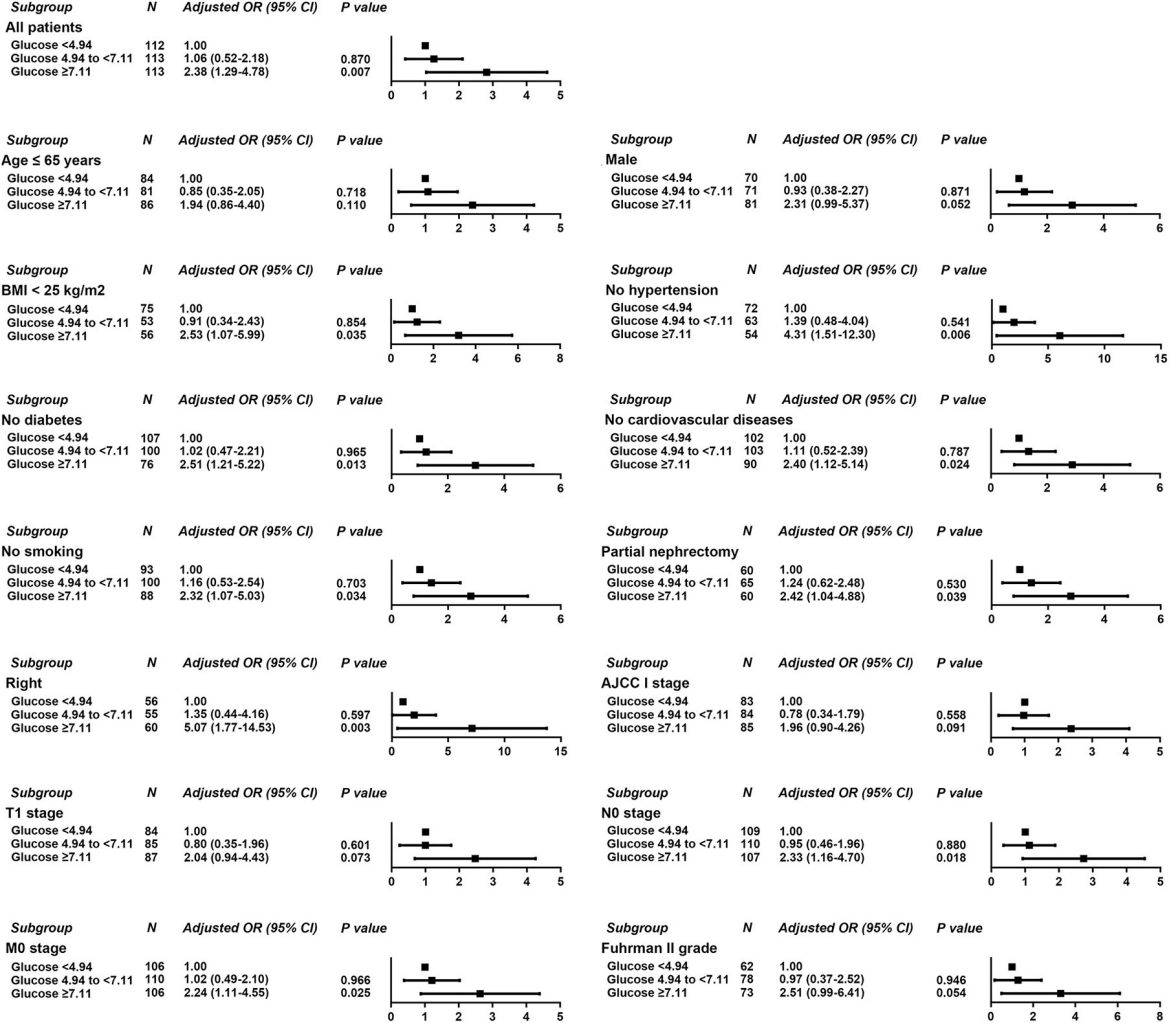
Results of subgroup analyses of POBG and a hospital length of stay > 3 weeks according to clinical characteristics.

Logistic regression was used to evaluate the relationship between POBG and hospital LOS > 2 weeks or > 3 weeks. We found that POBG was an independent risk factor for LOS > 2 weeks or > 3 weeks in both the univariate analysis, basic model, core model, and extended model. In the extended model, after adjusting for all variables, patients with POBG levels ≥ 7.11 mmol/L had a 115% higher risk of LOS > 2 weeks than patients with POBG levels <4.94 mmol/L (aOR 2.15; 95% CI 1.11-4.20; *p* = 0.024) and patients with POBG levels ≥ 7.11 mmol/L had a 129% higher risk of LOS > 3 weeks than patients with POBG levels <4.94 mmol/L (aOR 2.29; 95% CI 1.16-4.52; *p* = 0.017) (Figures 3, 4 and Table 4). Moreover, similar results were observed in the most subgroups analysis (Figures 3, 4).

**Table 4:** Relative risk of having a hospital LOS of >2 weeks or >3 weeks was calculated according to POBG level in tertile groups ^a^.

**Characteristic**	**N**	**Univariate analysis**		**Basic model**		**Core model**		**Extended model**	
		**aOR (95% CI)**	***P*-value**	**aOR (95% CI)**	***P*-value**	**aOR (95% CI)**	***P*-value**	**aOR (95% CI)**	***P*-value**
**LOS > 2 weeks**									
Glucose, mmol/L			**0.020**		**0.010**		**0.020**		**0.045**
Glucose <4.94	112	1.00		1.00		1.00		1.00	
Glucose 4.94 to <7.11	113	1.75 (0.99-3.11)	0.056	**1.92 (1.06**-**3.46)**	**0.030**	1.75 (0.99-3.11)	0.056	1.81 (0.99-3.31)	0.054
Glucose ≥ 7.11	113	**2.26 (1.24**-**4.11)**	**0.008**	**2.45 (1.33**-**4.52)**	**0.004**	**2.26 (1.24**-**4.11)**	**0.008**	**2.15 (1.11**-**4.20)**	**0.024**
**LOS > 3 weeks**									
Glucose, mmol/L			**0.001**		** <0.001**		**0.015**		**0.019**
Glucose <4.94	112	1.00		1.00		1.00		1.00	
Glucose 4.94 to <7.11	113	1.05 (0.54-2.04)	0.891	1.12 (0.57-2.21)	0.743	1.04 (0.52-2.09)	0.903	1.03 (0.51-2.08)	0.942
Glucose ≥ 7.11	113	**2.76 (1.51**-**5.07)**	**0.001**	**2.96 (1.59**-**5.51)**	**0.001**	**2.34 (1.19**-**4.58)**	**0.013**	**2.29 (1.16**-**4.52)**	**0.017**

a *Adjusted covariates: Basic model: age, gender and BMI; Core model: basic model plus hypertension, diabetes, cardiovascular diseases, smoking, ASA score and Clavien-Dindo complications; Extended model: core model plus surgery type, laterality, AJCC stage, T stage, N stage, M stage, and Fuhrman grade*.

*BMI, Body mass index; AJCC, American Joint Committee on Cancer; LOS, Length of stay; CI: confidence interval; aOR, adjusted odds ratio. P value < 0.05 are shown in bold*.

## Discussion

In this retrospective study, we included the clinical data of 338 patients who underwent laparoscopic nephrectomy in our center and explored the correlation between POBG levels and hospital LOS using univariate logistic regression, multifactor logistic regression, and dose-response analysis with restricted cubic spline functions. We found that POBG was an independent risk factor for hospital LOS. With the increase of POBG level, the risk of hospital LOS > 2 weeks or > 3 weeks increased, and there was a significant correlation between them in a non-linear dose-response manner.

An increasing number of studies have shown that abnormal blood glucose regulation was associated with the outcome of tumor patients. Kaneda et al. (14) found that poor glycemic control (HbA1c, ≥6.5%) was closely related to post-operative tumor recurrence in diabetic patients with hepatitis C virus-associated hepatocellular cancer undergoing curative resection. Lee et al. (15) retrospectively analyzed the clinical data of 746 prostate cancer patients undergoing radical prostatectomy (RP) and found that poor blood glucose control (HbA1c, ≥6.5%) was significantly associated with biochemical recurrence after RP. A recent study of patients with pancreatic neuroendocrine tumors found that pre-operative dysglycemia (blood glucose ≥ 140 mg% and/or HbA1c ≥ 6.5%) was independently associated with the recurrence-free survival (16). In addition, Liang et al. (17) also found that poor pre-operative blood glucose control (HbA1c, ≥7.0%) was significantly associated with an increased risk of recurrence and death in patients with cervical cancer who underwent radical hysterectomy. Our study found that high POBG was associated with the prolonged hospital LOS.

In this study, we found that patients in the high POBG group had a lower survival time compared to the other two groups. This may be due to patients in the high POBG group had higher proportion of hypertension, diabetes, cardiovascular disease, smoking, AJCC III/IV stage, T3-4 stage, N1 stage, and M1 stage than the other two groups, and patients in the high POBG group were more likely to have a combination of metabolic syndrome. Hyperglycemia can promote the production of advanced glycosylation end products, initiate lipid peroxidation and produce toxic aldehydes, which can cause damage to cellular DNA (18). Hyperglycemia can cause hyperinsulinemia and promote the epithelial-to-mesenchymal transition pathway, which promotes tumor cell metastasis (19). In addition, hyperglycemia can also inhibit the absorption of ascorbic acid by cells, which can cause damage to the immune system (20). Besides, chronic inflammation associated with metabolic syndrome can lead to the release of cytokines and further promote tumor growth (21). Moreover, hyperglycemia can increase the risk of infection at the surgical site and prolong wound healing time, which in turn increases the hospital LOS (10). We found that the optimal value of POBG is 5.66 mmol/L, which was below the clinical level. Based on the effect of blood glucose profile on LOS, our study suggests that patients' blood glucose profile should be corrected promptly if their POBG level is higher than 5.66 mmol/L. Our findings provide preliminary recommendations for optimizing blood glucose levels with or without comorbid diabetes. In addition, controlling lower POBG levels may delay the patient's hospital stay and has a guiding role in rapid recovery in the current era of fast-track surgery.

To our knowledge, this is the largest and first study of the relationship between POBG and hospital LOS in Chinese patients undergoing laparoscopic nephrectomy. However, there were several limitations of this study. First, this study is a single-center retrospective study, and further prospective studies of multiple health care systems are needed to verify its accuracy. Second, we did not include other treatments (immunotherapy, etc.) in the study, which will also have an impact on the LOS of patients. Due to the different surgeons, the discharge criteria of patients are also slightly different, which is a limitation of this study. In addition, with the development of technology and the accumulation of surgical experience, the LOS in recent years is shorter than that in previous years, which is also a limitation of this study.

## Conclusions

In summary, we found that higher POBG levels were significantly associated with prolonged LOS in patients with RCC undergoing laparoscopic nephrectomy.

## Data Availability Statement

The data analyzed in this study is subject to the following licenses/restrictions: The original dataset inquiries can be directed to the corresponding authors. Requests to access these datasets should be directed to Ming Chen, mingchenseu@126.com.

## Ethics Statement

The studies involving human participants were reviewed and approved by the methodology of this study followed the criteria outlined in the Declaration of Helsinki (as revised in 2013) and was ethically approved by the Ethics Committee and Institutional Review Board of Zhongda Hospital. The patients/participants provided their written informed consent to participate in this study.

## Author Contributions

TH, WM, WZ, WL, and MC: conception and design. BX, SC, and MC: administrative support. TH, WM, HL, TW, KP, and BX: collection and assembly of data. TH and WM: data analysis and interpretation. TH, WM, and CW: manuscript writing. All authors final approval of manuscript.

## Conflict of Interest

The authors declare that the research was conducted in the absence of any commercial or financial relationships that could be construed as a potential conflict of interest.

## Supplementary Material

Supplementary Figure 1 Relationship between post-operative blood glucose level and POBG.
