# Supplementary figures and images for: Relationship Between Pre-operative Blood Glucose Level and Length of Hospital Stay in Patients With Renal Cell Carcinoma Undergoing Laparoscopic Nephrectomy
Source: Front Surg. 2021 May 24;8:659365. doi: 10.3389/fsurg.2021.659365 (PMC8180589; doi:10.3389/fsurg.2021.659365)

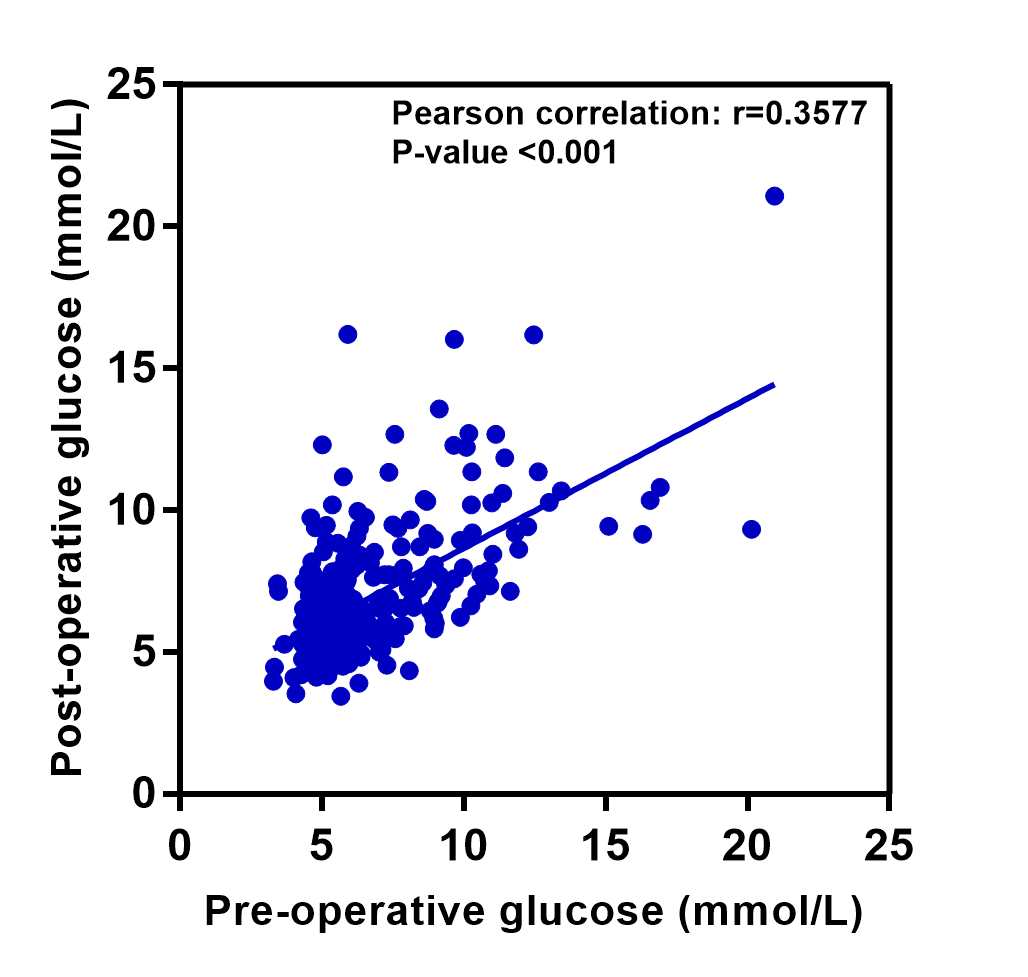

Supplement: Supplementary Figure 1 — Relationship between post-operative blood glucose level and POBG. [file Image_1.TIF]
